# Comparison of Brain White Matter Hyperintensities in Methamphetamine and Methadone Dependent Patients and Healthy Controls

**DOI:** 10.5812/iranjradiol.14275

**Published:** 2014-05-15

**Authors:** Abdulrasool Alaee, Mehran Zarghami, Samaneh Farnia, Mohammad Khademloo, Talayeh Khoddad

**Affiliations:** 1 Department of Radiology, Mazandaran University of Medical Sciences, Sari, Iran; 2 Department of Psychiatry, Mazandaran University of Medical Sciences, Sari, Iran; 3 Department of Community Medicine, Mazandaran University of Medical Sciences, Sari, Iran; 4 Department of Traditional Medicine, Mazandaran University of Medical Sciences, Sari, Iran

**Keywords:** Methamphetamine, Methadone, White Matter Hyperintensitiess, Magnetic Resonance Imaging

## Abstract

**Background:**

Previous studies have proven the development of white matter hyperintensities (WMH) in methamphetamine and opioid users. Opiates and methamphetamines (MA) are the most common addictive agents in Iran. The adverse effects of drugs on the CNS is of concern to specialists and researchers, and given that the neurotoxicity associated with methamphetamine is greater than opioids, it is hypothesized that the severity of WMH in patients with methamphetamine dependence is more than opioid drug-dependent individuals.

**Objectives:**

To our knowledge, this is the first research comparing the effect of methamphetamine and methadone (M) on the brain.

**Patients and Methods:**

In a historical cohort study, we compared WMH in the brain MRI of 50 methamphetamine-dependent patients, 50 methadone-dependent patients and 50 healthy volunteers who were matched for age, sex and dominant hand.

**Results:**

WMH was detected in 18 methamphetamine users, in 12 methadone users and in seven controls (P = 0.038). The site of brain lesions in MA users was mostly in the frontal lobe in 17 cases, in M users in the frontal lobe in 12 cases and in the control group, it was in the parietal lobe in four cases (P=0.001). The frontal lobes were the predominant locations of WMH in MA and M groups (P = 0.001). The frequency of brain lesions was mostly in the deep WM in 18 cases in MA users, in 12 cases in M users and in two cases in the control group (P=0.007). Hyper-signal foci of deep WM in the MA group were grade I (punctuate) in 12 cases, grade II (beginning confluence) in five cases and grade III (large confluent) in four cases. In the M group, there were six cases in grade I, three cases in grade II and one case in grade III. In the control group, there were three grade I cases, two grade II cases, and no grade III cases. Except for periventricular WMH (P = 0.13), there were statistical significant differences in the deep WMH (P = 0.007) and subcortex WMH (P = 0.01) between the three groups. The history of using other drugs and the duration of MA and M consumption were similar. The prevalence of brain lesions was generally higher in both drug user groups compared with the healthy controls. Increased WMH in the MA group was higher than the M group.

**Conclusions:**

A greater number of blood flow defects and ischemic lesions in the brain of MA users compared to opiate users may explain the prevalence of psychiatric disorders in these patients.

## 1. Background

Methamphetamine (MA) is a powerful central nervous system stimulant that strongly activates multiple systems in the brain (1). MRI is a very sensitive modality for evaluating the brain lesions, particularly in the white matter (WM). High signal lesions are observed in various parts of WM on T2WI, proton density and fluid attenuated inversion recovery (FLAIR) protocols. Neuropathologic causes of this phenomenon include:

1. expansion of the perivascular space,
2. perivascular demyelination,
3. astrocyte gliosis and
4. arteriosclerosis.

White matter hyper intensities (WMH) can be seen anywhere in the brain, such as sub-cortical regions, periventricular areas, basal ganglia and the brain stem (2).

WMH increases with cardiovascular risk factors such as older age, hypertension, and diabetes mellitus (3). The prevalence of WMH has been investigated in association with major depressive disorder (4) bipolar disorder (3) schizophrenia (4) and caffeine and nicotine effects (5). Previous studies have also demonstrated defects in brain perfusion resulting in ischemia among opiate consumers (4) and following MA use in animal (6) and human studies (7), and WMH in cocaine, MA and opiate users (3, 4, 8). Since MRI signals are not specific for WM lesions due to their similarity, determination of the location and shape of the lesions is necessary (2). Previous studies have demonstrated the presence of WMH in users of MA and opioid that are two of the most commonly used drugs in Iran. MA has a higher neurotoxicity than opioids and opiates cause both dilation of cerebral arteries through stimulation of nitric oxide and vasoconstriction, but MA is only vasoconstrictive (4). Based on the above-mentioned facts, it has been hypothesized that the severity of WMH in MA dependent patients is higher than opioid addicts.

## 2. Objectives

Hence, in this study we compared the WMH in the brain MRI of MA and M users with normal controls.

## 3. Patients and Methods

In a historical cohort study, the patients referred to the addiction clinic of Zare university hospital in Sari and also to private clinics in Sari and Qaemshahr, Mazandaran, Iran were studied. These were patients who were referred for treatment of addiction. A total of 50 MA-dependent patients, 50 M-dependent patients and 50 healthy volunteers were identified. Inclusion criteria were 19 to 49 years of age and the diagnosis of dependence based on the DSM-IV-TR criteria (9). Exclusion criteria included mental, medical and neurological illnesses that require immediate treatment, history of neurological diseases changing MRI findings, history of head trauma along with loss of consciousness for more than two minutes, abuse or dependence to other drugs at the time of study (except smoking), learning disability and mental retardation, visual impairment and sensorineural hearing disorders, diabetes mellitus, hypertension or ischemic cardiovascular disease, pregnancy and contraindications of MRI, including claustrophobia, metallic implants, and pace-maker (10).

Fifty age- and sex-matched healthy volunteer subjects were also recruited from Imam hospital. This healthy control group was selected based on the exclusion criteria and not having a history of substance abuse. The confounding variables such as age, sex and handedness were matched in three groups. After sample selection, the patients underwent brain MRI by General Electric model manufactured by USA with 1.5 Tesla power at Sari hospital. The axial fluid attenuation inversion recovery (FLAIR) images were produced based on the parameters of flip angle = 90°, FOV = 22 cm, TI = 2250 ms, TE = 145 ms and TR = 9900 ms and were reported by a radiologist who was unaware of the subject group. Classification of the lesions was conducted based on involvement of frontal, temporal, parietal, and occipital lobes. In addition, the lesions were classified based on the involvement of sub-cortical WM, deep parts of the WM, and periventricular WM. Hyper-signal foci were classified based on the intensity of the deep area of WM and periventricular WM according to the classification by Fazekas et al. (11): Grade 0: absence, grade I: punctuate foci, grade II: beginning confluence, and grade III: large confluent area.

The estimation of MA dosage consumption due to impurities was impossible, but in M users, it was an average of 110 mg per day. Statistical analysis was performed using SPSS version 19 for Windows software (SPSS Inc., Chicago, Ill, USA). Chi square analysis and fisher exact test were used to compare the qualitative variables while the quantitative variables were compared using independent t-test and one way ANOVA; ordinal logistic regression analysis was also used. In all cases P < 0.05 was considered as statistically significant. Written informed consent was obtained from all subjects. All aspects of the clinical research protocol were reviewed and approved by respective Institutional Review Boards.

## 4. Results

The mean age of the study sample was 36 ± 6.4 years (range: 21-49 years; 35.2 ± 3.1 in MA group, 37.4 ± 4.1 in M group and 34.4 ± 4.8 in controls, P-value = 0.9). As it has been shown in Table 1, the three groups were not significantly different in terms of age, sex, dominant hand, frequency of smoking and alcohol consumption in the past and during the study. The mean pack year of smoking was 16 ± 2 in the MA abuser, 14 ± 1.5 in the M abuser and 4 ± 1.2 in the control group. 

The level of SD (standard drink) was 28.12 ± 2.5 in MA abusers, 29.66 ± 3.9 in M abusers, and 4.2±0.8 in the control group (P = 0.001). The mean duration of drug use was also statistically similar between the two groups. A point to consider was the history of using other drugs along with M or MA; as among the M-dependents, 11.2% had a history of using opium, 11% had a history of using crystal, 4.7% had a history of using heroin kerack. In addition, among the MA-dependents, 21.24% mentioned a history of using opium, 13.5% a history of using crystal, 8% a history of using heroin kerack. However, none of the subjects was taking drugs other than M and MA at the time of the study and the control group had no history of any substance abuse. The past history of using other substances was not significantly different between the two groups of MA and M (P > 0.05). The mean duration of methamphetamine use in the MA group was about 26 ± 12.1 months and the mean duration of methadone use in the M group was 22 ± 8 months; the difference was not statistically significant. The mean duration of opium or heroin abuse (before MMT) was 18 ± 10 months and the mean duration of opioid abuse (before MMT+MMT) was 40 ± 18 months (P > 0.05).

**Table 1.** Demographic and Basic Characteristics of the Study Population ^a^

Variable	MA Group, No. (%)	M Group, No. (%)	Control Group, No. (%)	P Value
**Sex**				
Male	49 (98)	48 (96)	48 (96)	0.9
Female	1 (2)	2 (4)	2 (4)	
**Dominant Hand**				
Right	17	13	12	0.7
Left	3	2	4	
**Specific Underlying Disease**	-	-	-	
**Smoking History**	38 (76)	46 (92)	41 (82)	0.49
**Smoking During the Study**	12 (24)	9 (18)	14 (28)	0.49
**History of Alcohol Abuse**	8 (16)	6 (12)	5 (10)	0.65
**Alcohol Consumption During the Study**	7 (14)	2 (4)	3 (6)	0.24

^a^ Abbriviation: MA:methamphetamine; M: methadone

**Figure 1.**
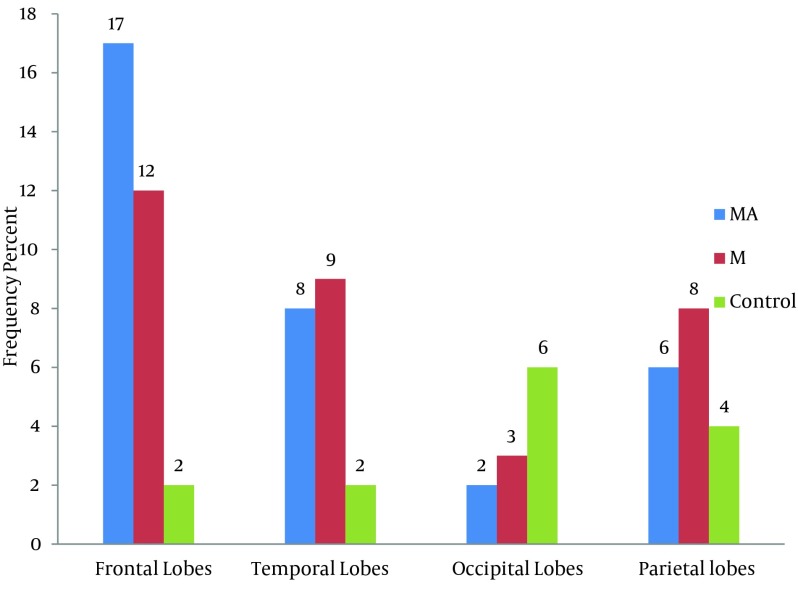
Comparison between the three groups based on the frequency of the lesion sites in MRI. MA, methamphetamine group; M, methadone group

According to Figure 1, the frequency of WMH foci in the brain MRI of the three groups demonstrated a statistically significant difference (P = 0.038). (P = 0.19, odds ratio = 1.78 (0.74-4.24) for M and MA groups, P = 0.011, odds ratio = 3.45 (1.28-9.25) for M and control groups and P = 0.2, odds ratio = 0.051 (0.18-1.44) for MA and control groups).

 The locations of WMH showed statistically significant differences in the frontal lobes (P = 0.001), but no statistically significant differences in the temporal lobes (P = 0.07), occipital lobes (P = 0.2) and parietal lobes (P = 0.07).

**Figure 2.**
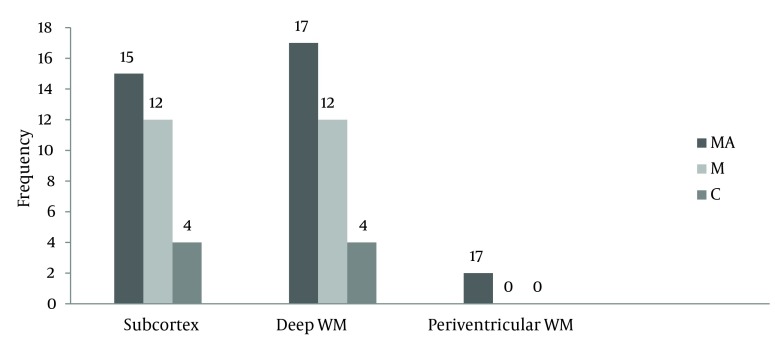
Comparison between the three groups based on the frequency of lesion sites in MRI in WM areas. MA, methamphetamine group; M, methadone group; C, control group.

**Figure 3.**
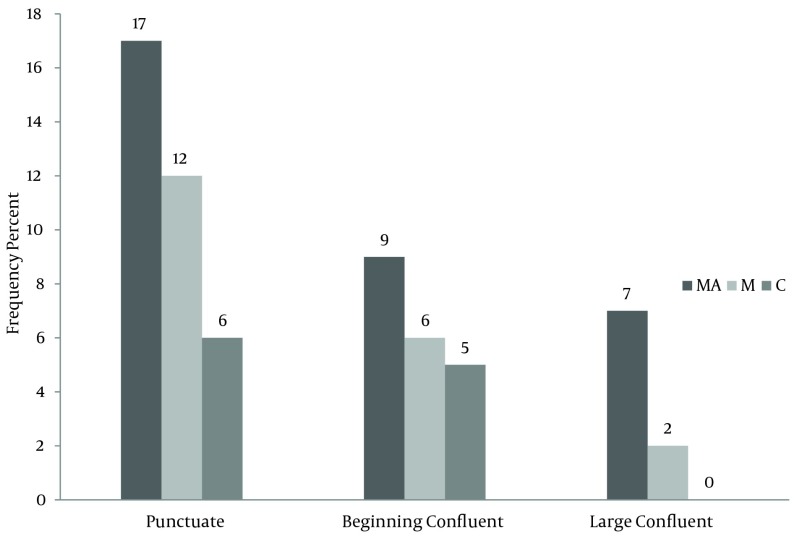
Comparison between the study subjects (frequency) based on the severity of brain lesions in the deep regions of WM and periventricular areas. MA, methamphetamine group; M, methadone group; C, control group.

**Figure 4.**
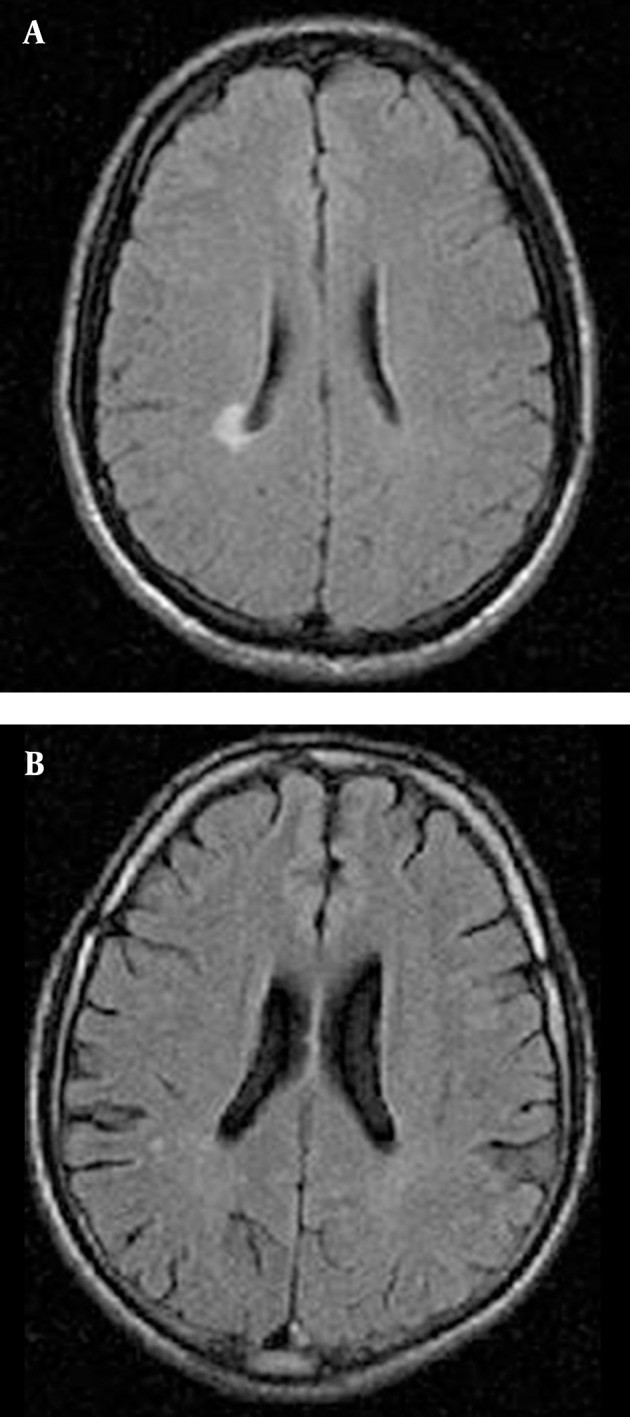
Axial FLAIR images. A) MA dependent subject, 27-year-old man with right paraventricular WM shows a large confluent area (GIII). B) Methadone dependent subject, 22-year-old woman, punctuate foci (GI) in the right parietal subcortex.

According to Figure 2, the frequency of brain lesions in MA and M user patients was more in deep WM areas compared with other regions based on the WMH, which showed a statistically significant difference (P = 0.007). These differences in the control group were not statistically significant (P = 0.13). In other words, the distribution of lesions in different parts of the brain was similar in the healthy controls. Classification of hyper-signal foci was also performed based on the intensity of the lesion in deep areas of MW as shown in Figure 3. In other words, the frequency of more severe lesions was higher in the MA group (P = 0.01). There was a statistically significant relationship between the location of the brain lesions and severity of the lesions (P < 0.001). Therefore, in those with simultaneous involvement of frontal and temporal lobes, the lesions were more severe. However, in 58.4% of the patients with involvement of these two cerebral lobes, the severity of the involvement was grade II and III in 74.6% of them. In general, among the study population, three cases of atrophy, one case of arachnoid cyst and one case of mild hydrocephalus were reported in MRI. In addition, one patient (2%) in the MA group had a lesion in the gray matter of subcortical areas of the brain with grade III severity and one patient (2%) in the M group had a lesion in the periventricular area with grade II severity. However, this difference was also not statistically significant (P = 0.46). As shown in Figure 4, representative images of high signal foci of the brain demonstrated the site and grades of severity in MA/M users.

## 5. Discussion

Distribution of brain lesions in different cerebral lobes was not similar. Lesions in the frontal lobe were more than the other parts in both MA and M groups. The frequency of brain lesions was significantly higher in the MA group compared with the M group, and in general, it was significantly higher in both drug user groups compared with the healthy controls.

To the best of our knowledge, this is the first report that systematically measured the rates and severity of deep, insular, and periventricular WMH in age- and sex-matched subjects with MA and M abuse compared to healthy subjects. These findings may explain prevalent psychiatric problems, particularly in MA users (12). In the present study, damage in the frontal lobe insular areas had the highest frequency followed by the deep WM in the MA group. This was significantly different compared with the M group.

Data analysis revealed a significant correlation between lesion severity and its location. Therefore, in those with frontal lobe involvement, the lesion severity was more as well. The toxic effects of MA on embryonic stem cell (ESC)-derived neuronal cells were also studied (13). Lyoo et al. found that the extent of tissue damage and brain lesions in the frontal and parietal lobes of heroin and cocaine dependent individuals was higher than the normal population and WMH lesions were located in frontal areas significantly (4). Chung et al. reported that in MA abusers, frontal WM integrity is compromised. This finding may also be related to the impairment in frontal executive function (14). Thompson et al. showed decreased cortical gray matter (mainly cingula), decreased volume of hippocampus and WM hypertrophy in the MRI of chronic users of MA (15). Coutinho et al. also reported changes in Golgi apparatus analysis. These molecular assessments demonstrated that type I interferons (interferon-alpha and interferon-beta) were increased in the frontal lobe (16).

Behavioral complications of MA use led researchers to pay more attention to this substance. In a study conducted by Wilson et al. dopamine, dopamine transporter, and tyrosine hydroxylase (dopamine-synthesizing enzyme) were reduced in chronic users of MA in human samples (17). Other imaging studies have also shown a decrease in vesicular monoamine transporter, dopamine receptors and N-acetylaspartate in MA users (18). Catecholaminergic and serotonergic nerve signaling may be altered by methamphetamine. Based on the theory, the emphasis is on differences in effects observed between the striatal and nucleus accumbens areas of the brain following acute as well as repeated dosing and withdrawal (19). Caffeine potentiates MA neurotoxicity, possibly via a mechanism involving an increase in dopamine release and excess reactive oxygen species generation (19).

Perfusion defects and ischemic lesions were demonstrated in the brain of MA users. By decreased oxygen supply to the brain tissue, MA causes ischemia and deep lesions in the WM of the brain (6). Functional MRI data analysis was used to demonstrate the related brain activation map, showing a high brain activation in the cingulate and low activation in the frontal lobe (20). A great deal of attention of neurotoxicity has been focused on reactive oxygen species (ROS) and reactive nitrogen species (RNS) as mediators of this toxicity (21). MA-induced hyperthermia, aberrant dopamine, and glutamate transmission; or mitochondrial disruption leads to the generation of reactive species with neurotoxic consequences (22). Brain hyperthermia may also result from metabolic activation induced by various addictive drugs, such as heroin, cocaine, and MA. MA increases brain metabolism, which is dose-dependent. It also diminishes heat dissipation because of peripheral vasoconstriction (23). Numachi et al. suggest that dopamine and serotonin transporters are the key molecules for hyperthermic and lethal toxic effects of MA (24).

Although the theory of vascular endothelial damage is one of the most well-known theories in the issue of brain tissue injury in these individuals (25) and also BBB integrity loss and permeability change has been recognized as a major cause of profound brain alterations (26), the exact mechanism of brain damage in MA users is still hypothetical. Of these hypotheses, toxic monoamine metabolites, glutamate-induced excitotoxic neurotoxicity, oxidative free-radical pathway, and metabolic stress can be noted (27). As hypothesized, the prevalence and severity of WMH were greater among MA users than opiate users. Probable factors that may explain this phenomenon include; first, MA has severe neurotoxicity and cerebral vasoconstriction with hyperthermia, but opiate induces both vasodilation and vasoconstriction; second, MA and opiate have different affects on dopamine, serotonin, norepinephrine and opiate receptors.

Hyperintensity signal changing is only observed in the WM of cerebral parenchyma, but not in the gray matter of the thalamus and basal ganglia. Although the etiology of this effect is not clear, it may be due to accumulation of MA, opiate and toxic substances in WM regions of the brain. Overall, the major findings of MA and opioid-induced neurotoxicity are neuronal loss, neurodegenerative alterations, reduction of glial fibrillary acidic protein-immunopositive astrocytes, widespread axonal damage with concomitant microglial activation as well as reactive and degenerative changes of the cerebral microvasculature. These observations demonstrate that MA and opioid initiate a cascade of interacting toxic, vascular and hypoxic factors, which finally result in widespread disturbances within the complex network of central nervous system cell-to-cell interactions (28). The mean age of the study population was 36.4 years that was similar to the previous studies, and as expected, drug use in men was significantly higher than in women. In the present study, the mean duration of drug use was generally 23 months, but no significant relationship was found between the duration of drug use and the severity of the lesions. It would had been better to assess the drug dosage at the time of the study, but there was a lot of recall bias. On the other hand, the used drugs, particularly MA, which were obtained from illicit resources, often had impurities that made the assessment of the actual amount of the effective drug unreliable. Any of the above-mentioned parameters might be the probable cause for no significant correlation between the severity of the lesions due to MA and its mean dosage. To explain the effect of gender on the brain lesions, it has been emphasized that estrogen has protective effects on inflammatory changes in the central nervous system of a woman in contrast to men (29), but based on the low number of women in this study, it was not possible to evaluate this issue. Chronic MA exposure is a contributor to the development of Parkinson's disease. There is a significant degree of striatal dopamine depletion at nigrostriatal dopaminergic neurons (30). Methadone overdose may induce toxic leukoencephalopathy (spongiform), but it is very rare (31).

There were limitations in this study. First, despite all efforts to match the main confounders, considering the large number of confounding factors, generalizing the findings of this study should be done with caution. In particular, it should be considered that people are rarely satisfied of using only one substance. Therefore, the similarity between the two groups regarding the "use of other drugs" and "duration of drug use" is not completely sufficient; "the type of other consumed drugs", "the amount of consumption" of each of them and their possible impurities as well as the current dosage of consumption affect the findings. Second, the smoking history in the addicted patients was mentioned as very heavy smokers (> 20 cigarettes). In smoking, the pack years are accurate and a standard variable of smoke exposure. The pack year variable is a term used to describe the number of cigarettes a person has smoked over time.

The pack year was calculated by averaging the number of cigarettes smoked daily, dividing by 20 (considered on pack) and multiplying by the number of years smoked (The pack year of smoking in MA abuser was 16 ± 2, 14 ± 1.5 in the M abuser, and 4 ± 1.2 in the control group). Fortunately, the number of alcohol consumers according to Islamic and social context is low, but they have a high SD (standard drink). A standard drink is a notional drink that contains a specified amount of pure alcohol (ethanol). The standard drink varies significantly from country to country. The SD of alcohol in consumers will vary according to the method of usage. 

The number of SD of a regular beer bottle with 5% alcohol is 1-1.5, a liquor bottle with 12-17% alcohol is 3.5-5 and a wine bottle with 50% alcohol is 30-40. It was difficult to calculate the SD of alcohol in subjects, because the majority of drug abusers used diluted ethanol (with water or juice) with above 90% alcohol (The level of SD in MA abusers was 28.12 ± 2.5, in M abusers it was 29.66 ± 3.9 and in the control group, this figure was 4.2 ± 0.8). 

In the present study, smoking and alcohol consumption before and during the study had no statistically significant association with severity, frequency or location of brain lesions among the MA or the M group of patients. Bae et al. also found no significant correlation when evaluating the possible effects of these two factors (P > 0.05) (3). Third, there is the small sample size of the female MA abusers. Fourth, comorbid drug use is a common confounder and it is not possible to find a majority of pure MA or M abusers. We hoped that enrolling a large number of subjects would in part compensate for different drug use profiles by individual subjects and provide meaningful data indicating relative severities of WMH in MA abuse vs. M abuse. Although an increased prevalence of WMH has been reported in a number of psychiatric disorders, it is not clear whether the cocaine and opiate abuses, which are associated with relatively high rates of psychiatric comorbidity, share the same pathophysiology for WMH with other psychiatric disorders.

In the MA group, the highest damage was seen in the frontal lobe, insular region and deep WM, which was different from the M group. The blood flow defects and ischemic lesions in the brain of MA users were greater than opiate users. Presence of these lesions can indicate nonviable areas of the brain after uptake of these neurotoxins.

## References

[1] Cottencin O, Rolland B, Guardia D, Karila L (2012). [Current data on methamphetamine].. Rev Prat..

[2] Maillard P, Delcroix N, Crivello F, Dufouil C, Gicquel S, Joliot M (2008). An automated procedure for the assessment of white matter hyperintensities by multispectral (T1, T2, PD) MRI and an evaluation of its between-centre reproducibility based on two large community databases.. Neuroradiology..

[3] Bae SC, Lyoo IK, Sung YH, Yoo J, Chung A, Yoon SJ (2006). Increased white matter hyperintensities in male methamphetamine abusers.. Drug Alcohol Depend..

[4] Lyoo IK, Streeter CC, Ahn KH, Lee HK, Pollack MH, Silveri MM (2004). White matter hyperintensities in subjects with cocaine and opiate dependence and healthy comparison subjects.. Psychiatry Res..

[5] Dager SR, Friedman SD (2000). Brain imaging and the effects of caffeine and nicotine.. Ann Med..

[6] Wang Y, Hayashi T, Chang CF, Chiang YH, Tsao LI, Su TP (2001). Methamphetamine potentiates ischemia/reperfusion insults after transient middle cerebral artery ligation.. Stroke..

[7] Chang L, Ernst T, Speck O, Patel H, DeSilva M, Leonido-Yee M (2002). Perfusion MRI and computerized cognitive test abnormalities in abstinent methamphetamine users.. Psychiatry Res..

[8] Goldstein RZ, Volkow ND (2002). Drug addiction and its underlying neurobiological basis: neuroimaging evidence for the involvement of the frontal cortex.. Am J Psychiatry..

[9] Compton WM, Dawson DA, Goldstein RB, Grant BF (2013). Crosswalk between DSM-IV dependence and DSM-5 substance use disorders for opioids, cannabis, cocaine and alcohol.. Drug Alcohol Depend..

[10] Hsu C, Parker G, Puranik R (2012). Implantable devices and magnetic resonance imaging.. Heart Lung Circ..

[11] Fazekas F, Chawluk JB, Alavi A, Hurtig HI, Zimmerman RA (1987). MR signal abnormalities at 1.5 T in Alzheimer's dementia and normal aging.. AJR Am J Roentgenol..

[12] Zarghami M (2011). Methamphetamine has changed the profile of patients utilizing psychiatric emergency services in Iran.. Iran J Psychiatry Behav Sci..

[13] Meamar R, Dehghani L, Karamali F (2012). Toxicity effects of methamphetamine on embryonic stem cell-derived neuron.. J Res Med Sci..

[14] Chung A, Lyoo IK, Kim SJ, Hwang J, Bae SC, Sung YH (2007). Decreased frontal white-matter integrity in abstinent methamphetamine abusers.. Int J Neuropsychopharmacol..

[15] Thompson PM, Hayashi KM, Simon SL, Geaga JA, Hong MS, Sui Y (2004). Structural abnormalities in the brains of human subjects who use methamphetamine.. J Neurosci..

[16] Coutinho A, Flynn C, Burdo TH, Mervis RF, Fox HS (2008). Chronic methamphetamine induces structural changes in frontal cortex neurons and upregulates type I interferons.. J Neuroimmune Pharmacol..

[17] Wilson JM, Kalasinsky KS, Levey AI, Bergeron C, Reiber G, Anthony RM (1996). Striatal dopamine nerve terminal markers in human, chronic methamphetamine users.. Nat Med..

[18] Chang L, Alicata D, Ernst T, Volkow N (2007). Structural and metabolic brain changes in the striatum associated with methamphetamine abuse.. Addiction..

[19] Sinchai T, Plasen S, Sanvarinda Y, Jaisin Y, Govitrapong P, Morales NP (2011). Caffeine potentiates methamphetamine-induced toxicity both in vitro and in vivo.. Neurosci Lett..

[20] Yin JJ, Ma SH, Xu K, Wang ZX, Le HB, Huang JZ (2012). Functional magnetic resonance imaging of methamphetamine craving.. Clin Imaging..

[21] Kuhn DM, Geddes TJ (2000). Molecular footprints of neurotoxic amphetamine action.. Ann N Y Acad Sci..

[22] Riddle EL, Fleckenstein AE, Hanson GR (2006). Mechanisms of methamphetamine-induced dopaminergic neurotoxicity.. AAPS J..

[23] Kiyatkin EA, Brown PL, Sharma HS (2007). Brain edema and breakdown of the blood-brain barrier during methamphetamine intoxication: critical role of brain hyperthermia.. Eur J Neurosci..

[24] Numachi Y, Ohara A, Yamashita M, Fukushima S, Kobayashi H, Hata H (2007). Methamphetamine-induced hyperthermia and lethal toxicity: role of the dopamine and serotonin transporters.. Eur J Pharmacol..

[25] Zhang X, Banerjee A, Banks WA, Ercal N (2009). N-Acetylcysteine amide protects against methamphetamine-induced oxidative stress and neurotoxicity in immortalized human brain endothelial cells.. Brain Res..

[26] Dietrich JB (2009). Alteration of blood-brain barrier function by methamphetamine and cocaine.. Cell Tissue Res..

[27] Kita T, Miyazaki I, Asanuma M, Takeshima M, Wagner GC (2009). Dopamine-induced behavioral changes and oxidative stress in methamphetamine-induced neurotoxicity.. Int Rev Neurobiol..

[28] Buttner A (2011). Review: The neuropathology of drug abuse.. Neuropathol Appl Neurobiol..

[29] Dluzen DE, McDermott JL (2004). Developmental and genetic influences upon gender differences in methamphetamine-induced nigrostriatal dopaminergic neurotoxicity.. Ann N Y Acad Sci..

[30] Thrash B, Thiruchelvan K, Ahuja M, Suppiramaniam V, Dhanasekaran M (2009). Methamphetamine-induced neurotoxicity: the road to Parkinson's disease.. Pharmacol Rep..

[31] Salgado RA, Jorens PG, Baar I, Cras P, Hans G, Parizel PM (2010). Methadone-induced toxic leukoencephalopathy: MR imaging and MR proton spectroscopy findings.. AJNR Am J Neuroradiol..

